# TDP1 deficiency sensitizes human cells to base damage via distinct topoisomerase I and PARP mechanisms with potential applications for cancer therapy

**DOI:** 10.1093/nar/gkt1260

**Published:** 2013-12-12

**Authors:** Meryem Alagoz, Owen S. Wells, Sherif F. El-Khamisy

**Affiliations:** ^1^Kreb's Institute, Department of Molecular Biology and Biotechnology, University of Sheffield, Sheffield, S10 2TN, UK, ^2^Genome Damage and Stability Center, University of Sussex, Falmer, Brighton, BN1 9RQ, UK and ^3^Center of Genomics, Helmy Institute, Zewail City of Science and technology, Giza, Egypt

## Abstract

Base damage and topoisomerase I (Top1)-linked DNA breaks are abundant forms of endogenous DNA breakage, contributing to hereditary ataxia and underlying the cytotoxicity of a wide range of anti-cancer agents. Despite their frequency, the overlapping mechanisms that repair these forms of DNA breakage are largely unknown. Here, we report that depletion of Tyrosyl DNA phosphodiesterase 1 (TDP1) sensitizes human cells to alkylation damage and the additional depletion of apurinic/apyrimidinic endonuclease I (APE1) confers hypersensitivity above that observed for TDP1 or APE1 depletion alone. Quantification of DNA breaks and clonogenic survival assays confirm a role for TDP1 in response to base damage, independently of APE1. The hypersensitivity to alkylation damage is partly restored by depletion of Top1, illustrating that alkylating agents can trigger cytotoxic Top1-breaks. Although inhibition of PARP activity does not sensitize TDP1-deficient cells to Top1 poisons, it confers increased sensitivity to alkylation damage, highlighting partially overlapping roles for PARP and TDP1 in response to genotoxic challenge. Finally, we demonstrate that cancer cells in which TDP1 is inherently deficient are hypersensitive to alkylation damage and that TDP1 depletion sensitizes glioblastoma-resistant cancer cells to the alkylating agent temozolomide.

## INTRODUCTION

It is becoming clear that human cells use distinct but functionally overlapping pathways to protect the genome from internal and external insults. Base damage and abasic (apurinic or apyrimidinic) sites ‘AP sites’ are common forms of DNA lesions that constitute ∼10^4^ lesions per cell per day (1). Base damage can be triggered endogenously in living cells as a result of base oxidation or from cofactors of biochemical reactions such as S-adenosylmethionine (2). Base damage can also result from the exposure to external alkylating agents such as fuel combustion products and tobacco smoke (3,4). AP sites are generated by the spontaneous or enzymatic hydrolysis of the N-glycosylic bond linking the damaged base to the deoxyribose sugar (5). The latter is conducted by monofunctional DNA glycosylases to remove damaged bases during base excision repair (BER) (6). AP sites can block progression of DNA and RNA polymerases, and if bypassed by translesion polymerases could result in base substitution and mutations (7,8). AP endonuclease 1 (APE1) maintains genetic integrity by hydrolysing the deoxyribose backbone at the 5′-side of the AP site, resulting in a nick with a 3′-hydroxyl and 5′-deoxyribose phosphate (5′-dRP), which are further processed by the short-patch or long-patch base excision repair [reviewed in (9,10)]. Cleavage of AP sites can also occur at the 3′-side through a β- or δ-elimination reaction initiated by dual function DNA glycosylases/lyases, resulting in a nick with 3′-α,β-unsaturated aldehyde (11,12). The resulting ‘dirty’ 3′- and 5′-DNA termini are restored to conventional 3′-hydroxyl and 5′-phosphate by a variety of DNA end-processing activities such as the 5′-dRP lyase activity of DNA polymerase β (Pol β), the endonuclease activity of flap endonuclease 1 or the phosphatase/kinase activity of polynucleotide kinase phosphatase [recently reviewed in (13)].

In addition to base damage and AP sites, another form of DNA lesion features proteins linked to DNA termini. It can arise during the normal enzymatic cycles of DNA topoisomerases where they form transient covalent linkage with the 3′-terminus of DNA (e.g. topoisomerase I ‘Top1’) or with the 5′-terminus (e.g. topoisomerase II ‘Top2’). These normal enzymatic cycles become abortive if the transient topoisomerase-DNA complex collides with DNA or RNA polymerases or in the presence of adjacent nicks, gaps or DNA secondary structures. Cells use specific enzymatic activities with distinct polarities to hydrolyze the covalent linkage between the stalled topoisomerase and DNA. This is typified by tyrosyl DNA phosphodiesterase 1 and 2 (TDP1 and TDP2), which remove Top1 and Top2 linked DNA breaks, respectively. The phosphodiesterase activity of TDP1 has also been implicated in processing other types of blocking 3′-lesions such as 3′-phosphoglycolates [recently reviewed in (14)]. More recently, biochemical studies using recombinant protein and cellular studies in *Schizosaccharomyces pombe* and chicken DT40 cells have suggested a role for TDP1 in processing AP sites and 3′dRP lesions (15–17). However, whether TDP1 protects human cells from base damage and the mechanisms by which it exerts this function are unknown. Here, using human MRC5 cells and cancer cell lines inherently deficient for TDP1 or resistant to alkylation-based chemotherapy, we show that TDP1 deficiency sensitizes human cells to base damage, independently of APE1. Top1 depletion alleviated the hypersensitivity to base damage, illustrating that alkylation-induced cytotoxicity is partly dependent on Top1. TDP1 promotes the repair of both Top1- and AP/3′-dRP lesions induced by alkylating agents via its tyrosyl DNA phosphodiesterase and AP/3′dRP lyase activities. Although the former is PARP dependent, the latter is PARP independent, pointing at new clinical settings for the emerging applications of PARP inhibitors.

## MATERIALS AND METHODS

### Cell culture and transfection

Human MRC5, DLD1, T98G, U87 cells were grown in minimum essential medium (Gibco) supplemented with 10% foetal bovine serum (PAA), 2 mM L-glutamine, 100 unit/ml penicillin and 100 U/ml streptomycin (Gibco) (18,19). RKO cells were grown in RMPI medium supplemented with 10% foetal bovine serum, 2 mM L-glutamine, 100 U/ml penicillin and 100 U/ml streptomycin. MRC5 cells were infected with Misson™ lentivirus particles (Sigma) containing shRNA targeting human TDP1 (5′-CCGGGCACGATCTCTCTGAAACAAACTCGAGTTTGTTTCAGAGAGATCGTGCTTTTTG-3′) or with GIPZ lentivirus particles (Thermo) containing shRNA targeting human topoisomerase I (5′-CCAGAGAATGTCAAGTTTT-3′). Control ‘non target’ shRNA lentivirus particles were used as a control (Sigma). Infection was conducted for 16 h in opti-MEM (Serum free, Gibco) without antibiotic followed by addition of fresh complete medium for 24 h. Stably infected cells were maintained in 1ug/µl puromycin (Sigma). APE1, TDP1 or Top1 transient knockdown was achieved using ON-TARGETplus Smart pool from Dharmacon. Sequences for TDP1 (CUAGACAGUUUCAAAGUGA, GACCAUAUCUAGUAGUGAU, UCAGUUACUUGAUGGCUUA, GGAGUUAAGCCAAAGUAUA), for Top1 (GAAAAUGGCUUCUCUAGUC, GAUUUCCGAUUGAAUGAUU, GCACAUCAAUCUACACCCA, CGAAGAAGGUAGUAGAGUC), for APE1 (CAAAGUUUCUUACGGCAUA, GAGACCAAAUGUUCAGAGA, CUUCGAGCCUGGAUUAAGA, UAACAGCAUAUGUACCUAA). Cells maintained in suspension at 2 × 10^4^ per 6-cm dish were transfected with 20 pM/ml siRNA in serum-free medium (Opti-MEM) containing Metafecten (Cambio). To improve knockdown efficiency, a second round of transfection was conducted after 24 h. Knockdown efficiency was determined 48–72 h after transfection using immunoblotting*.* For ectopic overexpression of TDP1 and APE1 in MRC5, cells grown to 50% confluency on 60-cm dishes were transfected with 1 μg empty mammalian expression construct or 1 μg of plasmids encoding human Myc-tagged TDP1, human Flag-tagged APE1 or both, using Metafecten transfection reagent (Cambio). Expression of the fusion proteins was analyzed 24 h following transfection and survival was conducted on the same samples used to assess protein expression, as described earlier in text.

Chicken DT40 cells, were cultured a 39°C and 5% CO_2_ in RPMI 1640 supplemented with 10% FCS, 1% chicken serum, 2 mM l-glutamine, 100 U/ml penicillin and 100 μg/ml streptomycin with 10^−^^5^ β-Mercaptoethanol. DT40 *Tdp1*−/− cells were described previously (20). For the generation of stable *Tdp1*−/− cells expressing human TDP1, 5 μg of linearized pCD2E vector containing G418 resistance cassette and 15 μg of linearized Myc-tagged hTDP1 (21) or empty vector were mixed with 5 × 10^6^ cells in 0.5 ml ice-cold PBS. Samples were then transferred to a Gene Pulser cuvette (Bio-Rad) with a 0.4-cm electrode gap and left for 10 min at 4°C, using the Bio-Rad Gene Pulser. Samples were then electroporated at 550 V and 25 μFD and chilled on ice for 10 min. Cells were then transferred to a 10-cm dish containing 20 ml pre-warmed complete medium and left overnight at 39°C. Cells were diluted in 80 ml media supplemented with 2 mg/ml G418 for selection and aliquoted into 200 μl samples in 4 × 96-well plates. Cells were then left for 7 days until colonies became visible. Approximately 2 mm diameter cells were transferred to 24-well plates with 1.5 ml of pre-warmed medium and left until they reached 100% confluency (∼3 days). Cells were then harvested and samples analyzed for stable clones by anti-Myc immunoblotting.

### Immunoblotting

Cells were lysed in 50 mM Tris–HCl, 500 mM NaCl, 2 mM EDTA, 2 mM EGTA, 25 mM NaF, 25 mM β-glycerolphosphate, 0.1 mM NaOrthovanadate, 0.2% Triton X-100, 0.3% NP-40, 10 U/ml of Benzonase nuclease and protease inhibitor cocktail (Roche) for 1 h at 4°C. Cellular debris was removed by centrifugation at 13 000 rpm for 15 min. Total protein concentration was determined by Bradford Assay, and equal concentrations were separated by 10% SDS–PAGE and transferred to nitrocellulose membranes. Membranes were blocked in 6% non-fat dry milk and incubated with primary antibodies overnight at 4°C. Horse-radish peroxidase (HRP)-conjugated secondary antibodies were incubated for 1 h at room temperature, and membranes were developed with enhanced chemiluminescence (Western lightening Plus-ECL, Perkin Elmer). Primary antibodies used were rabbit polyclonal anti-Tdp1 (1:500; MW: ∼75 kDa) developed in the laboratory by Eurogentec using TDP1^151^^−608^ as an antigen, mouse monoclonal anti-Top1 (1:1000; MW: ∼100 kDa) from Santa Cruz biotechnology (sc-32736), rabbit polyclonal anti-APE1 (1:1000; MW:∼37 kDa) from Novus Biologicals (NB100–101), anti-Tubulin (1:3000; MW: ∼55 kDa) from Abcam (ab4074), anti-MGMT (1:1000; MW:∼25 kDa) from Abcam (ab7045), anti-Myc (1:4000; 9B11; cell signalling), anti-PCNA (1:2000; MW: ∼29 kDa) from Alan Lehmann and anti-Actin (1:3000; MW:∼42 kDa) from Sigma-Aldrich (a3853). HRP-conjugated secondary antibodies were from Dako (1:3000; P0260 and P0448).

### Clonogenic survival and viability assays

Cells were plated in 10-cm dishes in duplicate and mock-treated with DMSO or with the indicated doses of camptothecin (CPT; Sigma) for 1 h, methyl methanesulfonate (MMS; Sigma) for 15 min, Temozolomide (TMZ; Sigma) at 37°C. Where indicated, cells were pre-incubated with 150 μM APE1 inhibitor CRT0044876 (Sigma) for 90 min (MRC5) or 120 min (cancer cell lines) or with 1 μM PARP inhibitor Olaparib for 60 min. Cells were washed 1× with phosphate-buffered saline and 2× with complete medium. Cells were then allowed to grow for 7–10 days until the formation of macroscopic colonies. Colonies were fixed in 80% cold ethanol and stained with 1% methylene blue. Survival was calculated by dividing the average number of colonies on treated plates by the average number of colonies on untreated plates. Data are the mean ± s.e.m. of three biological replicates.

DT40 cells (0.5 ml; 1 × 10^5^ cells in 10 ml) were mixed with 0.5 ml media containing increasing concentrations of CPT or MMS in the presence or absence of 0.5 μM PARP inhibitor Olaparib. Five hundred cells/well were seeded in triplicate into 96-well plates with 100 μl of medium/well. Plates were incubated at 39°C for 72 h and cell viability determined using the CellTiter-Blue kit (Promega). Briefly, 20 μl of CellTiter-Blue reagent was added to each well and fluorescence was measured at 560 nm Ex/590 nm Em using GloMax®-Multi Detection System (Promega) and data analyzed using SigmaPlot. Viability of untreated cells was set to 100% and error bars represent standard error from three independent biological repeats.

### Alkaline single-cell agarose gel electrophoresis assays

Cells were incubated with the indicated doses of CPT for 60 min or MMS for 15 min at 37°C. DNA strand breakage [primarily DNA single-strand breaks (SSBs) and alkali labile sites]were quantified by alkaline comet assays essentially as described (22). Briefly, cells were suspended in pre-chilled phosphate buffered saline (PBS) and mixed with equal volume of 1.2% low-gelling-temperature agarose (Sigma, Type VII) maintained at 42°C. Cell suspension was immediately layered onto pre-chilled frosted glass slides (Fisher) pre-coated with 0.6% agarose and maintained in the dark at 4°C until set, and for all further steps. Slides were immersed in pre-chilled lysis buffer [2.5 M NaCl, 10 mM Tris–HCl, 100 mM ethylenediaminetetraacetic acid (EDTA) (pH 8.0), 1% Triton X-100 and 1% dimethylsulfoxide (DMSO); (pH 10)] for 1 h, washed with pre-chilled distilled water (2 × 10 min) and placed for 45 min in pre-chilled alkaline electrophoresis buffer (50 mM NaOH, 1 mM EDTA and 1% DMSO). Electrophoresis was then conducted at 1 V/cm for 25 min, followed by neutralization in 400 mM Tris–HCl (pH 7.0) for 1 h. Finally, DNA was stained with Sybr Green I (1:10 000 in PBS) for 30 min. Average tail moments from 50 cells/sample were measured using Comet Assay IV software (Perceptive Instruments, UK). Data are the average ± s.e.m from three independent experiments. Statistical analyses were conducted using student *t*-test.

### Measurement of global RNA transcription

MRC5 cells were grown on coverslips for 48–72 h in complete media deprived of serum. The Click-iT RNA Alaxa Flour 488 Kit (Invitrogen) was used according to manufacture’s instructions to quantify global RNA transcription. Briefly, cells were treated with the indicated doses of CPT for 1 h or with MMS for 15 min and either harvested immediately after treatment or washed twice with serum-free medium and incubated in drug-free medium for a subsequent 3 h to allow for transcription recovery. Cells were incubated with 0.1 mM 5-ethynl uridine (EU) for 30 min in presence or absence of CPT or MMS to label newly synthesized RNA. Cells were washed with PBS, fixed with 3.7% formaldehyde for 15 min and permeabilized with 0.5% Triton-X100 for 20 min at room temperature. Cells were then incubated with Click iT reaction cocktail containing Click-iT additive and Alexa Flour azide 488 for 30 min at 37°C. Following washing with PBS containing 3% BSA, cells were mounted on glass slides using a DAPI containing antifade medium. RNA labeled with EU was then subjected to immunofluorescence analyses. Average fluorescence signal was quantified from 200 to 300 cells using Corel Photo Draw software.

## RESULTS

**Figure 1.**
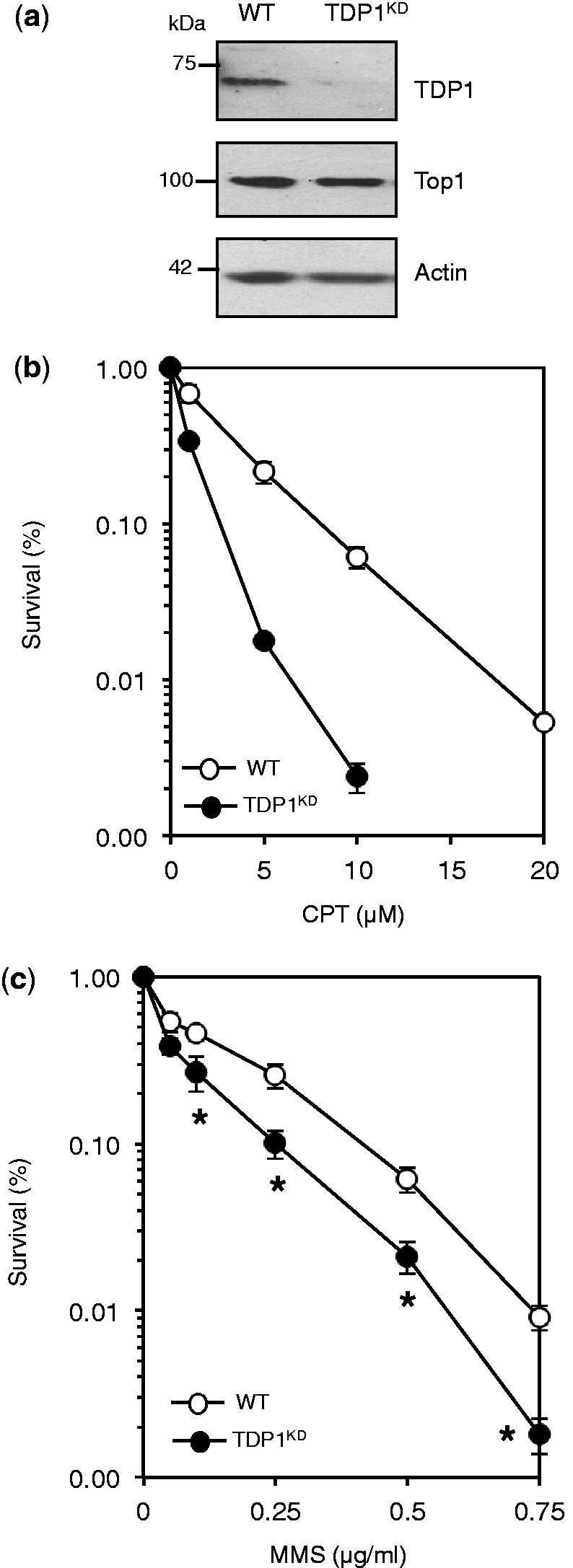
TDP1 promotes the repair of alkylation-induced base damage in human cells. (**a**) Human MRC5 cells were infected with lentivirus particles containing non-targeting control shRNA ‘WT’ or shRNA against human TDP1 ‘TDP1^KD^’. Cell lysates were fractionated by SDS–PAGE and analyzed by immunoblotting using anti-TDP1 (Eurogentec), anti-Top1 (Santa Cruz) or anti-actin (Sigma) immunoblotting. (**b**) WT or TDP1^KD^ MRC5 cells were incubated with the indicated concentrations of camptothecin ‘CPT’ for 60 min at 37°C and the ability of single cells to form macroscopic colonies was determined by dividing the average number of colonies on treated plates by the average number of colonies on untreated plates and presented as percentage survival. Data are the mean ± s.e.m. of three biological replicates. (**c**) Survival was determined in WT and TDP1^KD^ MRC5 cells following exposure to the indicated doses of the alkylating agent methyl methanesulfonate (MMS) for 15 min at 37°C as described in (a). Data are the mean ± s.e.m. of three biological replicates. Asterisks denote statistically significant difference between WT and TDP1^KD^ (*P* = 0.04, 0.001, 0.02, 0.002 for MMS doses 0.1, 0.25, 0.5 and 0.75 μg/ml, respectively; *t*-test).

To examine the role of TDP1 in response to DNA alkylation damage, we generated human MRC5 cells in which endogenous level of TDP1 was stably depleted using shRNA. TDP1 expression in cells infected with TDP1 shRNA was reduced by ∼90% compared with cells infected with control shRNA (Figure 1a). Consistent with a role for TDP1 in repairing Top1-mediated DNA damage, TDP1-depleted cells (TDP1^KD^) were more sensitive than controls to the Top1 poison camptothecin (CPT) (Figure 1b). To test whether TDP1 depletion confers hypersensitivity to base damage, we incubated control and TDP1^KD^ cells with increasing concentrations of the alkylating agent methyl methanesulfonate (MMS). MMS induced a dose-dependent killing in both cell lines, but cell death in TDP1^KD^ was significantly higher (*P* < 0.05; Figure 1c), illustrating a role for TDP1 in protecting human cells from base damage.

**Figure 2.**
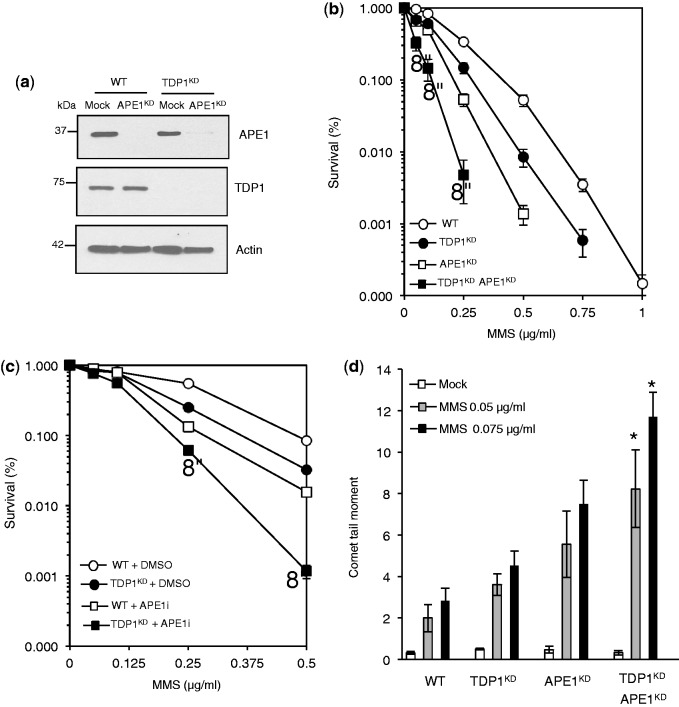
Human cells depleted for TDP1 and APE1 exhibit synergistic hypersensitivity to MMS. (**a**) WT or TDP1^KD^ MRC5 cells were subjected to control non-targeting siRNA ‘Mock’ or siRNA against human APE1. Cell extract was fractionated by SDS–PAGE and analyzed by immunoblotting using anti-APE1 antibodies (Novus). (**b**) Control human MRC5 ‘WT’, TDP1^KD^, APE1^KD^ or cells depleted for both APE1 and TDP1 ‘TDP1^KD^ APE1^KD^’ were incubated with the indicated concentrations of MMS and percentage survival calculated from three independent replicates. Data are the mean ± s.e.m. Asterisks denote statistical difference (*P* < 0.01, *t*-test) between APE1^KD^ and TDP1^KD^ APE1^KD^ cells (**c**) WT and TDP^KD^ cells were incubated with DMSO or 150 μM of the APE1 inhibitor CRT0044876 ‘APEi’ for 2 h followed by an additional incubation with the indicated concentrations of MMS for 15 min at 37°C. Cell survival was calculated from the average of three independent experiments ± s.e.m. Asterisks denote statistical difference (*P* < 0.02; *t*-test) between mock and APEi-treated TDP1^KD^ cells (**d**) MRC5 cells were incubated with the indicated doses of MMS for 15 min at 37°C and DNA SSBs and alkali labile sites quantified by alkaline comet assays. Average tail moment from 50 cells/sample was measured using Comet Assay IV software (Perceptive) and data are the average ± s.e.m. of three independent experiments. Asterisks denote statistical difference (*P* < 0.05; *t*-test) between APE1^KD^ and TDP1^KD^ APE1^KD^ cells.

AP endonuclease I (APE1) is the major nuclease for excising abasic sites resulting from alkylation damage, and thus plays a key role in protecting human cells from this type of DNA breakage. To test whether TDP1 functions together in the same pathway as APE1, we transiently depleted APE1 in control and in TDP1^KD^ cells using siRNA and compared the ability of cells to survive MMS damage. Consistent with published data (23–25), depletion of ∼90% of cellular APE1 (Figure 2a) led to ∼10-fold reduction of the cellular resistance to MMS (Figure 2b). Notably, co-depletion of APE1 and TDP1 led to enhanced cell sensitization to MMS above that observed for depletion of TDP1 or APE1 alone, suggesting that TDP1 operates in a parallel pathway to APE1, to repair MMS-induced DNA damage. This was further confirmed by pharmacological inhibition of APE1 repair activity using CRT0044876 (26,27) (Figure 2c). These observations are consistent with cellular studies in *S. pombe* where *tdp1^−^apn2^−^* double mutant cells showed synergistic increase in MMS sensitivity compared with single mutants (16). To test whether the lyase (either AP, 3′-dRP or both) activity of TDP1 could explain, at least in part, the sensitivity of TDP1^KD^ cells to MMS, we measured DNA strand breaks and abasic sites using alkaline single-cell gel electrophoresis (comet assays). We postulated that the monofunctional alkylating agent MMS would produce base alkylation, which would undergo spontaneous or glyosylase-mediated base release to generate cytotoxic abasic sites. The alkaline nature of the comet assay would uncover abasic sites resulting from defects in APE1 or TDP1. Similarly, processing of AP sites in cells and generation of 3′-dRP lesions would also result in detectable SSBs by the alkaline comet assay. As predicted, depletion of APE1 led to ∼2.5-fold increase of SSBs (Figure 2d). Although depletion of TDP1 led to an increase of MMS-induced SSBs, co-depletion of TDP1 and APE1 led to higher number of SSBs than depletion of either enzyme alone.

**Figure 3.**
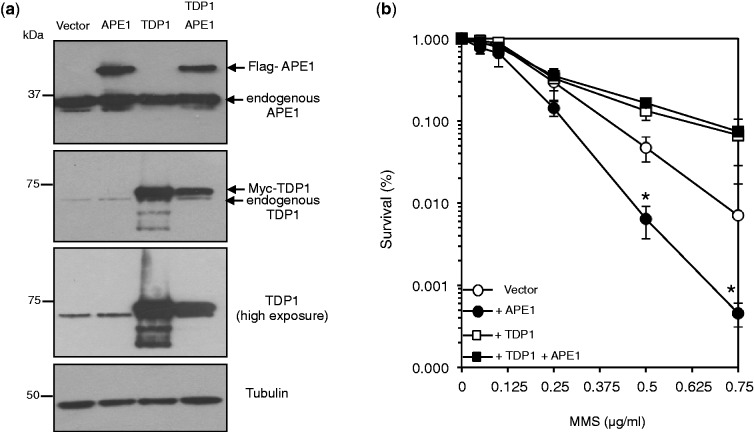
Overexpression of TDP1 protects human cells from MMS-induced DNA damage. (**a**) MRC5 cells were transfected with an empty mammalian expression vector ‘Vector’, or constructs encoding Flag-APE1, Myc-TDP1 or both. Expression of fusion proteins was analyzed by fractionating cell lysates using SDS–PAGE and immunoblotting, using anti-TDP1 (Eurogentec), anti-APE1 (Novus) or anti-tubulin (Sigma) antibodies. (**b**) MRC5 ectopically expressing Flag-APE1, Myc-TDP1 or both was incubated with the indicated concentrations of MMS and percentage survival calculated from three independent replicates. Data are the mean ± s.e.m. Asterisks denote statistical difference (*P* < 0.01;*t*-test) between cells overexpressing APE1 alone and cells expressing both APE1 and TDP1.

It is worth noting that 3′-dRP lesions are more cytotoxic than AP sites, and thus more likely to account for the survival data (Figure 2b), as they would not be bypassed by translesion polymerases. We, therefore, hypothesized that overexpression of APE1 would generate excessive toxic intermediates that exceeds the ability of downstream repair factors to deal with, causing cellular hypersensitivity to MMS. If it is true that TDP1 protects from APE1-induced lesions, either directly or indirectly, then its overexpression would confer protection. To test this hypothesis, we generated MRC5 cells in which APE1 and TDP1 were ectopically overexpressed separately or together, and compared their sensitivity to MMS (Figure 3a). APE1 overexpression sensitized MRC5 cells to MMS, which is consistent with published work (28) and, importantly, increasing the cellular pool of TDP1 offered a remarkable protection (Figure 3b and Supplementary Figure S1). Together, these data demonstrate that TDP1 protects human cells from MMS damage.

**Figure 4.**
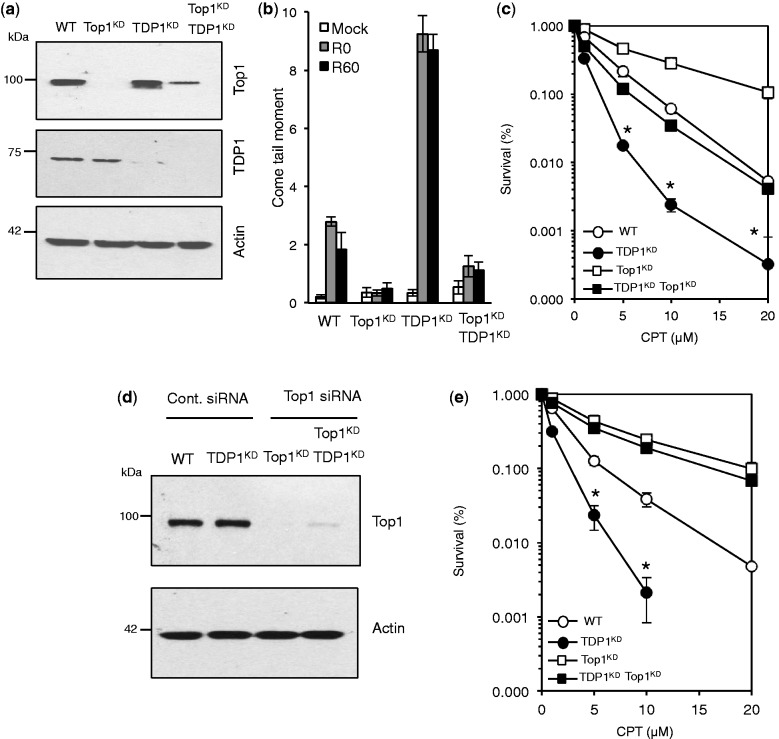
Depletion of Top1 protects human cells from camptothecin-induced cell death. (**a**) Control ‘WT’ or TDP1^KD^ human MRC5 cells were subjected to lentivirus particles containing control non-targeting shRNA or shRNA against human Top1 ‘Top1^KD^’ to generate stable cells in which TDP1 or Top1 was depleted separately or together. Cell lysates were fractionated by SDS–PAGE and analyzed by immunoblotting using anti-TDP1 or anti-Top1 antibodies. (**b**) The indicated cell lines were incubated with 20 μM CPT for 1 h at 37°C and DNA strand breakage quantified using alkaline comet assays. Average tail moments from 50 cells/sample were measured, and data are the average ± s.e.m. of three independent experiments. (**c**) The indicated MRC5 cells were incubated with increasing concentrations of CPT for 60 min at 37°C and survival was determined from three biological replicates and presented as mean ± s.e.m. (**d**) Cells were additionally subjected to control siRNA or Top1 siRNA to deplete residual levels of Top1 in TDP1^KD^/Top1^KD^ cells and cell lysate analyzed by SDS–PAGE and immunoblotting. (**e**) Control ‘WT’, TDP1^KD^, Top1^KD^ or TDP1^KD^/Top1^KD^ cells in which Top1 level was additionally depleted by siRNA were treated with increasing concentrations of CPT for 60 min at 37°C and survival determined from three biological replicates and presented as mean ± s.e.m. Asterisks denote statistical difference (*P* < 0.05; *t*-test) between TDP1^KD^ and TDP1^KD^ Top1^KD^ cells.

How does TDP1 fulfil cellular protection from alkylation damage? DNA nicks and gaps have been shown to trap Top1 on DNA resulting in Top1 single-strand breaks (Top1-SSBs), and the ability of TDP1 to process these structures (29) may contribute to its protective role in response to MMS. To test whether this is the case, we generated human cells in which TDP1 and Top1 were depleted separately or together (Figure 4a). We first examined the accumulation of Top1-SSBs following exposure to CPT (Figure 4b). TDP1^KD^ cells accumulated ∼5-fold more Top1-SSBs than control cells while additional depletion of Top1 prevented their accumulation (Figure 4b). Furthermore, Top1 depletion protected TDP1^KD^ cells from cell death induced by CPT to levels comparable with those observed in control cells (Figure 4c). We noted, however, that depletion of Top1 did not fully protect TDP1^KD^ cells from CPT as it did for control cells. We reasoned this may be due to ∼25% residual Top1 expression in Top1^KD^/TDP1^KD^ double mutants compared with almost complete Top1 depletion in single Top1^KD^ cells (Figure 4a). Additional depletion of Top1 using siRNA led to marked reduction of Top1 expression (Figure 4d) and increased the CPT resistance of Top1^KD^/TDP1^KD^ double mutants to levels observed in single Top1^KD^ cells (Figure 4e).

**Figure 5.**
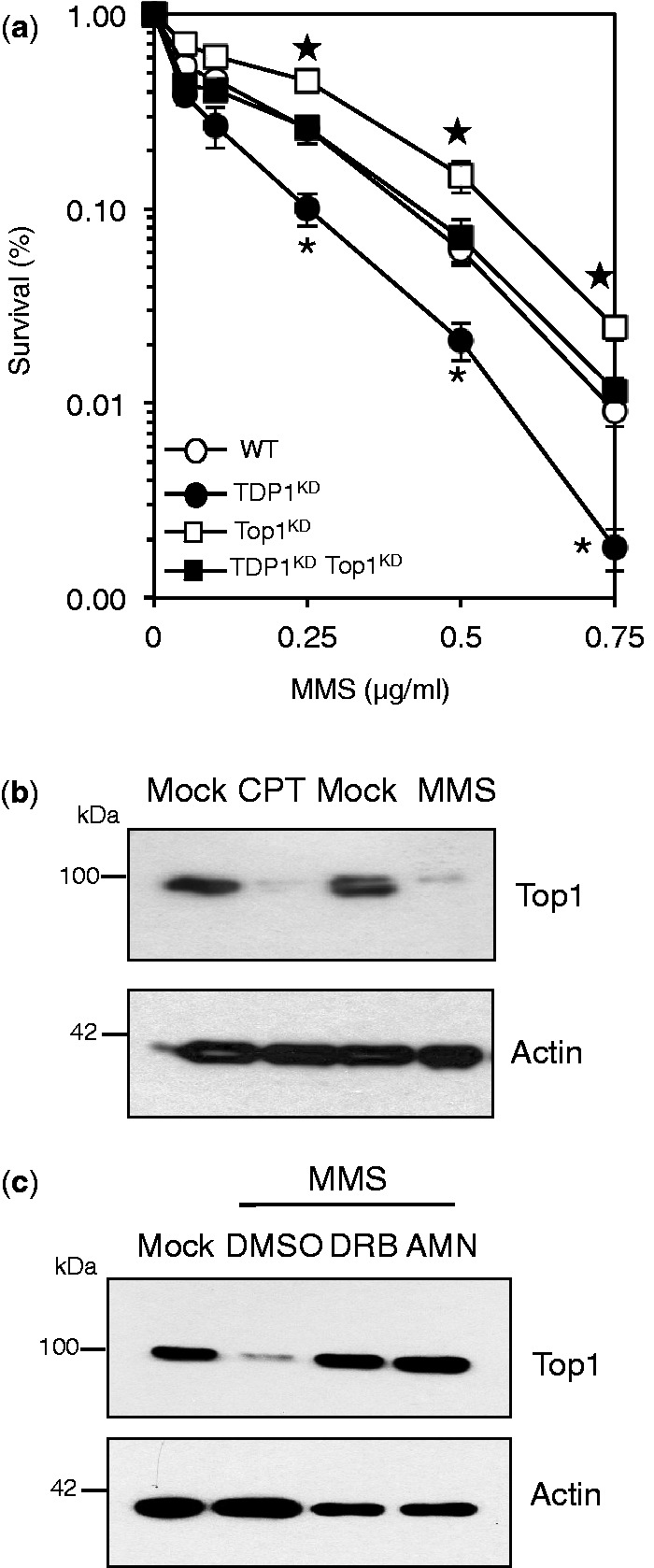
Depletion of Top1 protects human cells from alkylation-induced DNA damage. (**a**) Control ‘WT’, TDP1^KD^, Top1^KD^ or TDP1^KD^/Top1^KD^ double mutant cells in which Top1 level was additionally depleted by siRNA were incubated with increasing concentrations of MMS for 15 min at 37°C. Survival was determined from three biological replicates and presented as mean ± s.e.m. Top1 depletion protects TDP1^KD^ from MMS damage (Asterisks; *P* < 0.05; *t*-test between TDP1^KD^ ‘closed circles’ and TDP1^KD^ Top1^KD^ cells ‘closed squares’). This protection did not fully restore resistance to levels observed in Top1^KD^ cells (Stars; *P* < 0.01; *t*-test between Top1^KD^ ‘open squares’ and TDP1^KD^ Top1^KD^ cells ‘closed squares’), suggesting that a proportion of MMS-induced breaks are processed by TDP1 in a Top1-independent manner. (**b**) Human MRC5 cells were incubated with 20 μM CPT or with 2 μg/ml MMS for 3 h at 37°C and cell lysate fractionated by SDS–PAGE and analyzed by immunoblotting. (**c**) MRC5 cells were incubated with DMSO or 50 μM 5,6-dichloro-1-β-D-ribofuranosylbenzimidazole ‘DRB’ for 1 h or with 1 μg/ml α-amanitin for 16 h followed by an additional 3 h incubation with 2 μg/ml MMS. Cell lysates were fractionated by SDS–PAGE and analyzed by immunoblotting.

Having now established a cellular system to specifically study Top1-SSBs in response to genotoxic stress, we next examined their contribution to the cytotoxicity conferred by MMS. To our surprise, depletion of Top1 conferred remarkable protection in TDP1^KD^ cells from MMS damage (Figure 5a), suggesting that Top1-breaks contribute to MMS-induced cytotoxicity. If it is true that MMS induces Top1-breaks, then persistent exposure to MMS would result in Top1 degradation similar to persistent exposure to CPT. Incubation of MRC5 cells with CPT or MMS led to significant depletion of Top1 (Figure 5b). As is the case for Top1 poisons, MMS-induced Top1 degradation was largely dependent on transcription since pre-incubation with two different transcription inhibitors 5,6-dichloro-1-beta-D-ribofuranosylbenzimidazole (DRB) or α-amanitin largely reduced or ablated Top1 degradation (Figure 5c). Together, these data suggest that alkylation damage generates transcription-associated Top1-breaks, which contributes to TDP1’s protective role in response to MMS.

**Figure 6.**
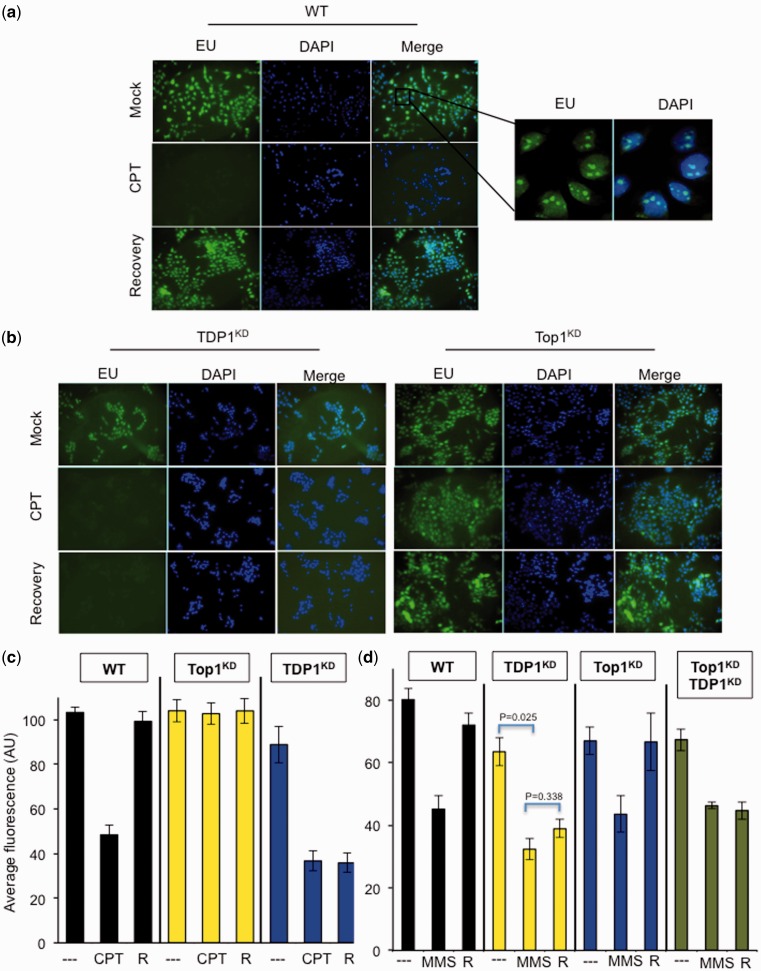
TDP1 promotes transcription recovery following alkylation-induced DNA damage. (**a**) Control MRC5 cells ‘WT’ grown on coverslips were maintained for 2 days in serum-free media, treated with DMSO ‘Mock’ or with 20 μM CPT ‘CPT’ for 1 h and either harvested immediately after treatment or incubated in CPT-free media for a subsequent 3 h to allow for transcription recovery. Cells were incubated with 0.1 mM 5-ethynl uridine (EU) for 30 min to label newly synthesized RNA, which was visualized by using the Click iT reaction with Alexa Flour azide 488. EU-labeled RNA was subjected to immunofluorescence analyses and DNA counterstained with 4',6-diamidino-2-phenylindole (DAPI). Representative micrographs are depicted. (**b**) TDP1^KD^ or Top1^KD^ MRC5 cells were examined for nascent RNA synthesis as described in (a). (**c**) Average fluorescence signal (arbitrary units ‘AU’) from 200 to 300 cells as described in (a) and (b) were quantified from three biological replicates ± s.e.m. (**d**) The indicated MRC5 cells were grown in serum-free medium and treated with 2 μg/ml MMS for 15 min at 37°C. Cells were either harvested immediately after treatment ‘MMS’ or incubated in MMS-free media for a subsequent 1 h ‘R’. Newly synthesized RNA was quantified and average fluorescence signal of EU-labeled RNA was quantified as described earlier from three biological replicates.

As DNA nicks, gaps and Top1-breaks are predicted to block progression of elongating RNA polymerases, we next examined whether the protective role conferred by TDP1 in response to alkylation damage can be explained by its role during transcription. For these experiments, we first examined transcription competence following CPT in serum-arrested human cells, to minimize the impact of DNA replication. Incubation of control cells with CPT led to remarkable reduction of global transcription, which was restored to background levels following subsequent incubation in CPT-free media (Figure 6a). Consistent with a role for TDP1 to repair transcription-blocking Top1-breaks (21), its depletion led to a reduction in transcription that failed to recover during 2 h incubation in CPT-free media (Figure 6b, c). Importantly, transcription decline in these experiments was owing to Top1-DNA breaks because Top1-depeletion ablated the CPT-induced transcription decline.

We next examined transcription recovery following MMS. Similar to CPT, incubation of control cells with MMS reduced global transcription, which was restored to background levels following incubation in MMS-free media. Interestingly and consistent with a role for TDP1 in response to alkylation damage, TDP1-deficient cells failed to restore background transcription levels following MMS (Figure 6d). However, in striking contrast to CPT, MMS treatment of Top1-deficient cells (Top1^KD^) also led to a comparable reduction in transcription to that observed in Top1 proficient cells, suggesting that MMS induces transcriptional arrest via a Top1-indepenedent mechanism, presumably via the ability of AP sites and 3′dRP lesions to block progression of RNA polymerases. Consistent with this, additional depletion of TDP1 in Top1^KD^ cells led to a similar reduction in transcription, which was not restored to background levels during MMS-free recovery periods. We conclude from these experiments that TDP1 promotes transcription recovery following MMS and suggest that this is due to its AP/3′-dRP lyase activity.

**Figure 7.**
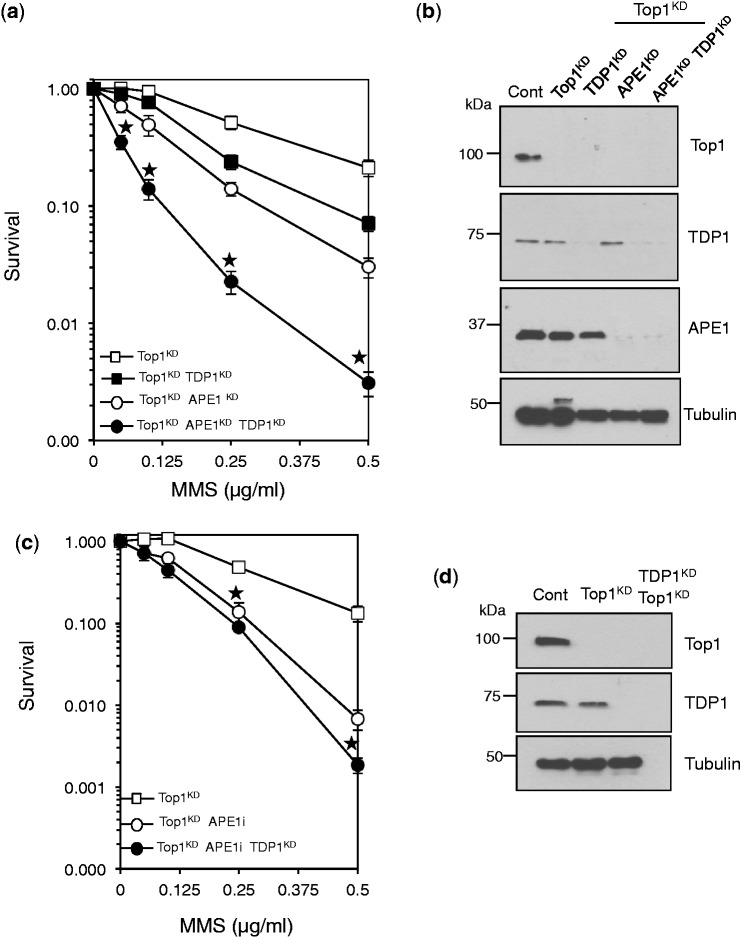
Depletion of TDP1 sensitizes Top1^KD^/APE1^KD^ cells to alkylation-induced DNA damage. (**a**) Top1^KD^ cells were subjected to control siRNA or siRNA for TDP1 or APE1, separately or together and cells were incubated with increasing concentrations of MMS for 15 min at 37°C. Survival was determined from three biological replicates and presented as mean ± s.e.m. Asterisks; *P* < 0.05; *t*-test between Top1^KD^/TDP1^KD^ ‘open circles’ and Top1^KD^/TDP1^KD^/APE1^KD^ ‘closed circles’. (**b**) Lysates from cells described in ‘a’ were fractionated by SDS–PAGE and analyzed by immunoblotting. (**c**) Top1^KD^ or Top1^KD^/TDP1^KD^ cells were incubated with DMSO or 150 μM of the APE1 inhibitor CRT0044876 ‘APEi’ for 2 h followed by an additional incubation with the indicated concentrations of MMS for 15 min at 37°C. Cell survival was calculated from the average of three independent experiments (two biological replicates) ± s.e.m. Asterisks denote statistical difference (*P* < 0.05; *t*-test) between APEi treated Top1^KD^ and Top1^KD^/TDP1^KD^ cells. (**d**) Lysates from cells described in ‘c’ were fractionated by SDS–PAGE and analyzed by immunoblotting.

To what extent do TDP1’s phosphodiesterase and lyase activities contribute to the protective effect from MMS? As both activities use the same active site (17), it was difficult to generate a separation-of-function mutant. We reasoned that eliminating the contribution of TDP1 phosphodiesterase activity on MMS-induced Top1-breaks by depleting Top1, coupled with limiting APE1 lyase function, would allow us to directly examine the lyase role of TDP1. Any sensitization induced by TDP1 knockdown in this system would be due to its lyase and not phosphodiesterase activity. Pharmacological inhibition of APE1 or its siRNA-mediated depletion sensitized Top1^KD^ cells to MMS (Figure 7a, b), reflecting the importance of APE1 lyase to protect from MMS. Importantly, additional depletion of TDP1 led to marked sensitization (compare open and closed circles Figure 7a, b). Together these observations suggest a role for TDP1 lyase in response to MMS damage, particularly at conditions where APE1 is limiting.

**Figure 8.**
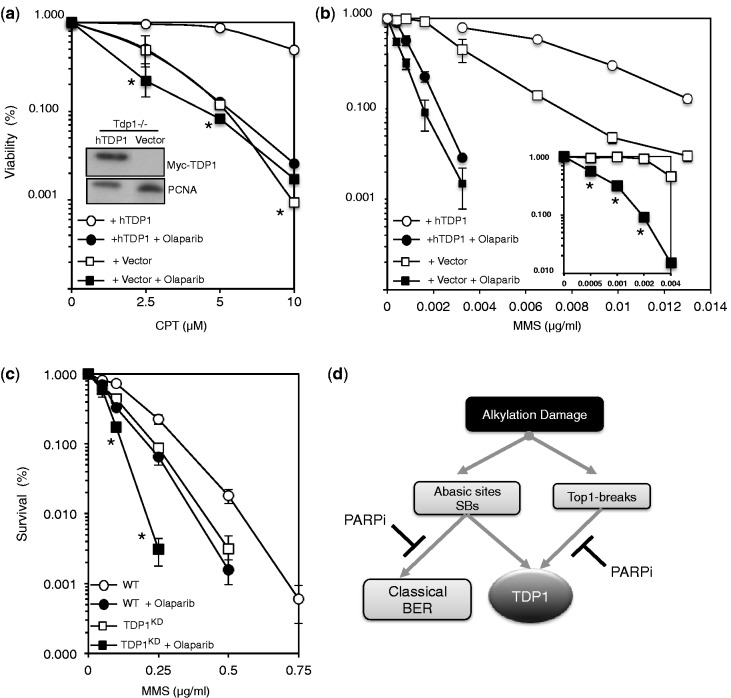
The PARP inhibitor Olaparib sensitizes TDP1-deficient cells to alkylation damage but not Top1 poisons. (**a**) Chicken DT40 Tdp1−/− cells were stably transfected with an empty mammalian expression vector ‘Vector’ or with a construct expressing human Myc-TDP1 ‘hTDP1’. Cells were pre-incubated with 0.5 μM of the PARP inhibitor Olaparib for 1 h at 39°C followed by additional incubation of the indicated concentrations of CPT for 72 h at 39°C. Viability was determined by quantifying fluorescence signals using CellTiter-Blue. Viability of untreated cells was set to 100% and error bars represent standard error from three independent biological repeats. Inset: DT40 cell lysate fractionated by SDS–PAGE and analyzed by anti-Myc (9B11; Cell signaling) and anti-PCNA (PC10) immunoblotting. Asterisks denote statistical differences (*P* > 0.1; *t*-test) between Vector alone ‘open squares’ and Vector + Olaparib ‘closed squares’ (**b**) Viability of the indicated DT40 cells was analyzed in presence or absence of Olaparib as described earlier in text, following incubation with the indicated concentrations of MMS for 72 h. Data are the average of three independent repeats ± s.e.m. Asterisks denote statistical differences (*P* < 0.02; *t*-test) between Vector alone ‘open squares’ and Vector + Olaparib ‘closed squares’ Inset: survival curves as described earlier in the text following incubation with a lower dose range of MMS (**c**) WT and TDP1^KD^ human MRC5 cells were pre-incubated with 1 μM Olaparib for 60 min followed by additional incubation with the indicated concentrations of MMS for 15 min at 37°C. Survival was determined from three independent repeats and data are the average ± s.e.m. Asterisks denote statistical differences (*P* < 0.01, *t*-test) between TDP1^KD^ ‘open squares’ and TDP1^KD ^+ Olaparib ‘closed squares’ (**d**) Model for the repair of alkylation-induced DNA damage by TDP1. Base damage induces both Top1- and AP/3’-dRP DNA breaks. The former are dealt with TDP1 in a PARP-dependent process, whereas the latter are processed in a PARP-independent mechanism. PARP activity appears to promote TDP1 alternative pathways in response to alkylation damage, which likely involves canonical base excision repair factors such as APE1.

To further dissect the mechanisms by which TDP1 protects from MMS damage, we exploited our observations in chicken DT40 cells where inhibition of Poly (ADP-ribose) polymerase (PARP) activity did not further sensitize Tdp1−/− cells to Top1 poisons (Figure 8a). Inhibition of PARP has been shown to impair DNA SSB repair due to trapping of the enzyme at repair intermediates, physically blocking subsequent repair events (30). We reasoned that if the cytotoxicity of TDP1-deficient cells to MMS is mediated solely via Top1-breaks then PARP inhibition would not confer additional sensitization, as is the case for Top1 poisons. In a remarkable contrast to this proposition, Tdp1−/− cells treated with the PARP inhibitor Olaparib were significantly more sensitive to MMS than untreated cells (Figure 8b). This was also confirmed in human MRC5 cells in which TDP1 levels were stably depleted using shRNA (Figure 8c). These data suggest that PARP and TDP1 operate together to repair Top1-breaks but independently to repair AP/dRP breaks (Figure 8d).

**Figure 9.**
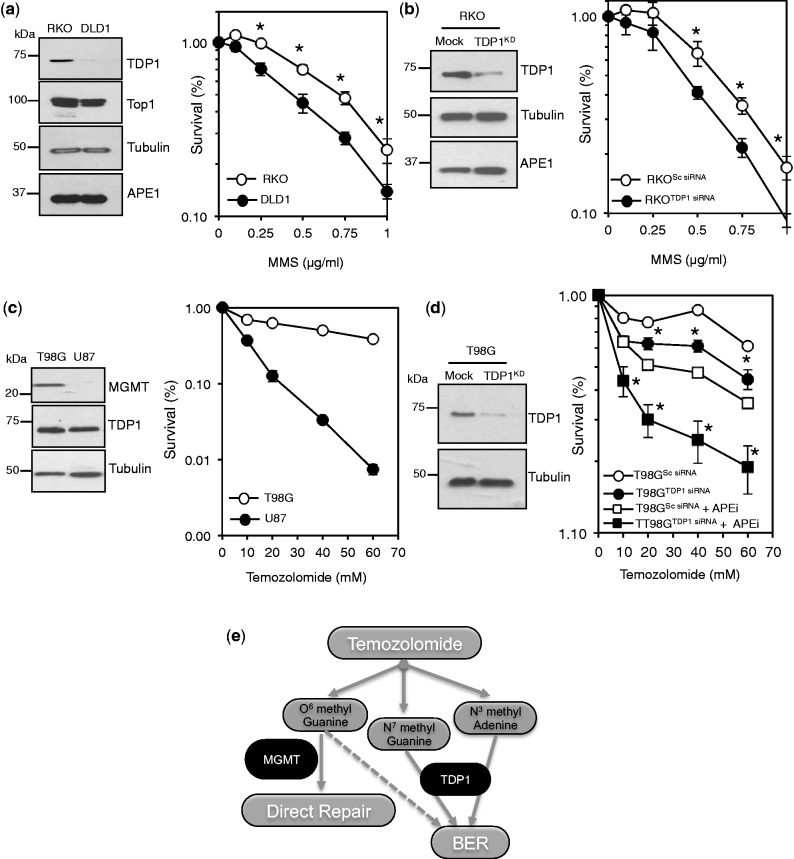
TDP1 depletion sensitizes glioblastoma-resistant cancer cells to temozolomide. (**a**) Cell lysates from RKO and DLD1 cancer cell lines were fractionated by SDS–PAGE and analyzed by immunoblotting using anti-TDP1 (Eurogentec), anti-Top1 (Santa Cruz), anti-Tubulin (Abcam) and anti-APE1 (Novus) antibodies (left). RKO and DLD1 cells were compared for their survival following exposure with the indicated doses of MMS for 15 min at 37°C (right). Data are the average of three independent experiments ± s.e.m. Where not visible, error bars are smaller than the symbol. (**b**) RKO cells were subjected to scrambled siRNA (Mock) or siRNA against TDP1 (TDP1^KD^) and cell lysates analyzed by immunoblotting (left). Control RKO (RKO^Sc siRNA^) and RKO cells in which TDP1 levels were depleted (RKO^TDP1 siRNA^) were examined for their survival following exposure to the indicated doses of MMS, as described earlier in the text (right). (**c**) Glioblastoma multiforme T98G and U87 cancer cell lines were analyzed for methylguanine methyltransferase (MGMT) expression using anti-MGMT antibodies (Abcam) (left). High-MGMT expressing T98G cells were compared with low-MGMT expressing U87 cells for their survival following exposure to increasing concentrations of the alkylating agent temozolomide (right). (**d**) TG98 cells were subjected to scrambled siRNA (Mock) or siRNA against TDP1 (TDP1^KD^) and cell lysates analyzed by immunoblotting (left). Control T98G cells (T98G^Sc siRNA^) and cells in which TDP1 levels were depleted (TG98^TDP1 siRNA^) were incubated with DMSO or 150 μM of the APE1 inhibitor CRT0044876 ‘APEi’ for 2 h followed by additional incubation with the indicated concentrations of MMS for 15 min at 37°C. Cell survival was blindly scored from three independent biological repeats, and data are the average ± s.e.m. (right). (**e**) Model for the repair of temozolomide-induced DNA breaks. Temozolomide delivers a methyl group to purine bases of DNA, resulting in O^6^-methyl guanine, N^7^-methyl guanine and N^3^-methyl adenine. The primary cytotoxic lesion is believed to be O^6^-methyl guanine, which can be removed by a direct repair mechanism mediated by methylguanine methyltransferase (MGMT). Inherent and acquired resistance to temozolomide via MGMT expression presents a major challenge in cancer therapy, particularly for glioblastoma multiforme. TDP1 promotes the repair of methylated purines induced by temozolomide via a distinct non-canonical BER pathway. Increasing the load of unrepaired methylated purines by exploiting the limited availability of TDP1 alone or in combination with canonical BER factors such as APE1 provides a new synthetic lethal setting to improve the clinical outcome of temozolomide-based cancer therapy. Asterisks denote statistical differences (*P* < 0.05; *t*-test) between control and TDP1-deficient cells.

Finally, to examine whether the involvement of TDP1 in MMS damage could be applied to improve alkylation-based chemotherapy, we screened a panel of cancer cell lines for TDP1 expression. We identified RKO and DLD1 colorectal cancer cells that express remarkably different levels of TDP1 (Figure 9a, left) and then compared their ability to survive MMS damage. Consistent with our data, DLD1 cells were more sensitive than RKO to MMS (Figure 9a, right). This hypersensitivity was not due to different levels of APE1 or Top1 (Figure 9a) and was not due to the non-isogenic nature of the two cell lines, as depletion of TDP1 using siRNA in RKO also led to marked sensitization to MMS (Figure 9b). Alkylation-based chemotherapy such as Temozolomide (TMZ) are widely used to treat a variety of human cancers, most notably Glioblastoma Multiforme (GM), which is the most common aggressive adult primary tumor of the central nervous system. TMZ delivers a methyl group to purine bases of DNA, resulting in O^6^-methyl guanine, N^7^-methyl guanine and N^3^-methyl adenine. The primary cytotoxic lesion is believed to be O^6^-methyl guanine, which can be removed by a direct repair mechanism mediated by methylguanine methyltransferase (MGMT). Inherent and acquired resistance to TMZ via MGMT expression present major obstacles in the clinical management of GM. In light of the data presented above, we reasoned that reducing TDP1 levels might offer a new tool to sensitize TMZ-resistant GM cells to alkylation damage. Consistent with previous work (31), T98G GM cells expressing MGMT exhibited significantly higher levels of resistance to TMZ than U87 cells that express undetectable levels of MGMT (Figure 9c). Depletion of TDP1 or the additional depletion of APE1 led to marked sensitization of T98G cells to TMZ (Figure 9d), suggesting the importance of determining and/or manipulating TDP1 expression levels to improve the clinical outcome of TMZ-based chemotherapy.

## DISCUSSION

Accumulating evidence suggest that human cells use distinct but functionally overlapping pathways to handle the endogenous and external load of DNA damage. As cancer cells are often perturbed in many DNA repair pathways (32), understanding the complexity of functional redundancy is key in exploiting the inherent biological variations in cancer. This study reveals a synergistic sensitivity of cells depleted for TDP1 and APE1 to alkylating agents, raising the possibility that TDP1 inhibitors could selectively target tumors with perturbed APE1 expression. In addition, the administration of alkylating agents in cancer cells in which TDP1 is inherently deficient, or in combination with TDP1 inhibitors, may also provide a new anti-cancer strategy to target specific class of tumors, particularly those that are resistant to alkylation damage such as temozolomide-resistant GM. In contrast to APE1 inhibition, TDP1 suppression is a more attractive tool, as it is predicted to provide a better tolerance during chemotherapy. This is suggested by gene knockout experiments in vertebrate systems where TDP1 deletion, unlike that of APE1, is not essential for organismal survival (33–35).

TDP1 was first identified as an activity in *S. cerevisiae* that is capable of cleaving the covalent phosphodiester linkage between a tyrosine residue in Top1 and 3′-phosphate on DNA (36). This type of DNA linkage is typically formed during abortive Top1 reactions and further work has shown that TDP1 protects mammalian cells from Top1-mediated DNA damage (33,37). The 3′-leaving group does not seem to be restricted on tyrosine because biochemical studies reported a decent activity of TDP1 on 3′-phosphoglycolate (38), AP site mimics (17,39) and 3′-dRP (15,16). The 3′-dRP lyase activity has been reported for human and *S. pombe* TDP1 where, in the latter, it presents the major APE1-independent activity. It is likely that TDP1 AP/dRP lyase is more important in yeast than in human due to the lack of NEIL proteins in yeast, which provide an extra layer of redundancy in dealing with AP sites in human cells (40). However, experiments in DT40 cells (15) and our observations here, showing a protective role of TDP1 independently of Top1 suggest a role for its lyase activity in human cells. Thus, despite its weaker activity on AP/dRP compared with canonical 3′-phosphotyrosyl substrates (15), we suggest that TDP1 AP/dRP lyase has a role in human cells, particularly if APE1 is limiting.

In addition to their use in the clinic to kill cancer cells, alkylation damage is almost unavoidable and it is intriguing to speculate that the progressive accumulation of AP/3′-dRP lesions may also contribute to neurological decline observed in TDP1-deficient patients with spinocerebellar ataxia with axonal neuropathy (SCAN1) (41). Accumulation of Top1-breaks during transcription has been associated with the neurological decline in SCAN1 and in other cerebellar degenerative hereditary disorders such as ataxia telangiectasia (42). However, the Top1-independent nature of transcriptional decline observed here (Figure 6d) suggests that the role of TDP1 during transcription in response to base damage is likely due to its lyase rather than its tyrosyl DNA phosphodiesterase activity.

One of the important observations of the current study is the demonstration that Top1 depletion protects mammalian cells from alkylation damage. Biochemical analyses have shown that Top1 can be trapped by a variety of endogenous DNA lesions including AP sites and strand breaks (43,44). Lethal DNA double-strand breaks (DSBs) can be generated by replication runoff of Top1-SSBs during S-phase, which likely contributes to the cell killing observed in this study following MMS. It is thus possible that in addition to its 3′-dRP/AP lyase (16,45) human TDP1 can protect from base damage via its ability to disjoin Top1 peptides from DNA. Intriguingly, the former activity appears to be PARP independent, as PARP inhibition further sensitized TDP1-deficeint cells to MMS, whereas it did not for CPT. It is likely that PARP and TDP1 function together in the same pathway to limit DSB formation and replication runoff following Top1 poisoning (46). We propose a model where PARP activity coordinates fork stabilization with the removal of stalled Top1 peptide from DNA termini by TDP1. This dual action of PARP may provide sufficient time to repair Top1-breaks by slowing or reversing the forks while promoting the resolution of Top1-breaks and preventing DSB formation. On the other hand, PARP inhibitors may trap PARP on a subset of MMS-induced repair intermediates, uncoupling them from subsequent repair and generating toxic lesions at replication forks that are distinct from those processed by TDP1 (47), thereby causing synergistic lethality. It is possible that the latter breaks are sufficiently different in origin, structure or accessibility to repair than those processed by PARP-related mechanisms and that enzymes acting on them are somehow excluded from acting on the other distinct class of breaks. Much remains to be determined on how these two pathways are prioritized in cells and whether one pathway predominates under particular circumstances. Nevertheless, these observations provide a new mechanistic rationale for the synergy of combining PARP inhibitors and Top1 poisons in clinical trials (46,48) and suggest the limited clinical utility of TDP1 in this context but highlights its potential importance during PARP inhibitor and TMZ combined therapy.

In summary, these data demonstrate a role for TDP1 in response to alkylation damage in human cells and illustrates the potential application of this knowledge to improve alkylation-based cancer therapy. TDP1 promotes the repair of both Top1- and AP/3′-dRP breaks induced by alkylating agents via its tyrosyl DNA phosphodiesterase and AP/3′dRP lyase activities. Although the former is PARP dependent the latter is PARP independent, opening new treatment paradigms for the emerging clinical applications of PARP inhibitors.

## SUPPLEMENTARY DATA

Supplementary Data are available at NAR Online.

## FUNDING

This work is funded by a Wellcome Trust fellowship [085284] and grant [091043 to S.E-.K.] whose lab is supported by a Lister Institute of Preventative Medicine Fellowship. Funding for open access charge: Wellcome Trust.

*Conflict of interest statement*. None declared.

## Supplementary Material

Supplementary Data
