# Supplementary material for: TDP1 deficiency sensitizes human cells to base damage via distinct topoisomerase I and PARP mechanisms with potential applications for cancer therapy
Source: Nucleic Acids Res. 2013 Dec 12;42(5):3089–103. doi: 10.1093/nar/gkt1260 (PMC3950670; doi:10.1093/nar/gkt1260)
Supplement: Supplementary Data [file supp_42_5_3089__index.html]

TDP1 deficiency sensitizes human cells to base damage via distinct topoisomerase I and PARP mechanisms with potential applications for cancer therapy — TDP1 deficiency sensitizes human cells to base damage via distinct topoisomerase I and PARP mechanisms with potential applications for cancer therapy — Supplementary Data 

# TDP1 deficiency sensitizes human cells to base damage via distinct topoisomerase I and PARP mechanisms with potential applications for cancer therapy

## Supplementary Data

files

**Files in this Data Supplement:**

- Supplementary Data - pdf file
