# Supplementary material for: TDP1 deficiency sensitizes human cells to base damage via distinct topoisomerase I and PARP mechanisms with potential applications for cancer therapy
Source: Nucleic Acids Res. 2013 Dec 12;42(5):3089–103. doi: 10.1093/nar/gkt1260 (PMC3950670; doi:10.1093/nar/gkt1260)
Supplement: Supplementary Data [file supp_gkt1260_nar-01684-d-2013-File011.pdf]

# Supplementary Figure 1

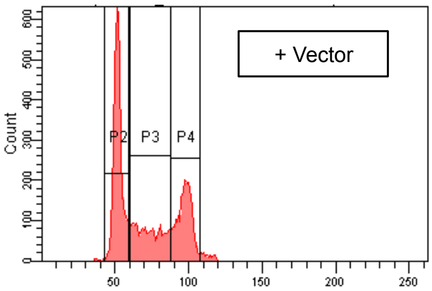


| Population | #Events | %Parent | %Total |
|---|---|---|---|
| All Events | 10,000 | | 100.0 |
| P1 | 8,150 | 81.5 | 81.5 |
| P2 | 3,737 | 45.9 | 37.4 |
| P3 | 2,082 | 25.5 | 20.8 |
| P4 | 2,241 | 27.5 | 22.4 |

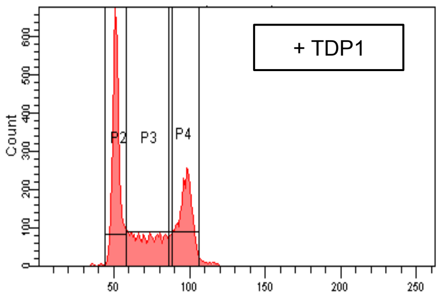


| Population | #Events | %Parent | %Total |
|---|---|---|---|
| All Events | 10,000 | | 100.0 |
| P1 | 8,242 | 82.4 | 82.4 |
| P2 | 3,400 | 41.3 | 34.0 |
| P3 | 2,259 | 27.4 | 22.6 |
| P4 | 2,612 | 31.7 | 26.1 |

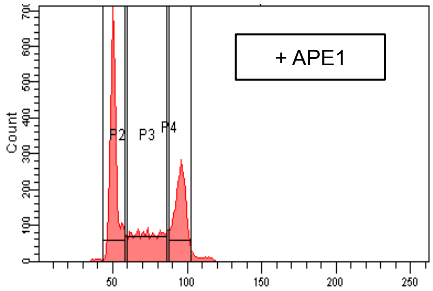


| Population | #Events | %Parent | %Total |
|---|---|---|---|
| All Events | 10,000 | | 100.0 |
| P1 | 8,000 | 80.0 | 80.0 |
| P2 | 3,282 | 41.0 | 32.8 |
| P3 | 1,920 | 24.0 | 19.2 |
| P4 | 2,348 | 29.3 | 23.5 |

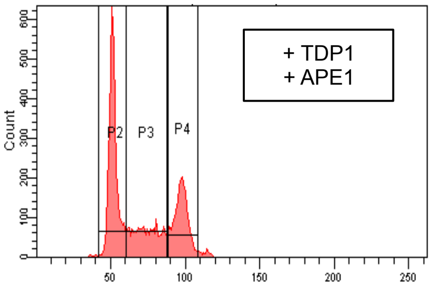


| Population | #Events | %Parent | %Total |
|---|---|---|---|
| All Events | 10,000 | | 100.0 |
| P1 | 7,288 | 72.9 | 72.9 |
| P2 | 3,377 | 46.3 | 33.8 |
| P3 | 1,745 | 23.9 | 17.4 |
| P4 | 1,889 | 25.9 | 18.9 |

**Suppl Fig.1. Overexpression of TDP1 or APE1 did not affect the cell cycle profile.** MRC5 cells were transfected with empty vector, Flag-APE1, Myc-TDP1,or both. Cells were fixed in 70% ethanol at 4°C overnight and stained with 10 µg/ml propidium iodide and 0.5 mg/ml RNase in PBS for 30 min. Cell cycle profile was determined using FACScan (Beckmann Coulter).
